# Improved Detectability of *Plasmodium falciparum* Clones with Repeated Sampling in Incident and Chronic Infections in Burkina Faso

**DOI:** 10.4269/ajtmh.21-0493

**Published:** 2021-11-01

**Authors:** Aissata Barry, Shehu S. Awandu, Alfred B. Tiono, Lynn Grignard, Teun Bousema, Katharine A. Collins

**Affiliations:** ^1^ Centre National de Recherche et de Formation sur le Paludisme (CNRFP), Ministère de la Santé, Ouagadougou, Burkina Faso;; ^2^ Department of Medical Microbiology and Radboud Center for Infectious Diseases, Radboud University Medical Centre, Nijmegen, The Netherlands;; ^3^ Department of Immunology and Infection, London School of Hygiene and Tropical Medicine, London, United Kingdom

## Abstract

We evaluated the detectability of *Plasmodium falciparum* clones when assessed on 3 consecutive days in incident and chronic infections in naturally exposed children living in an area of intense malaria transmission in Burkina Faso. The median number of clones by merozoite surface protein 2 (MSP2) genotyping was 3 (interquartile range [IQR] 2–5) in incident infections compared with 6 (IQR 4–8) in chronic infections (*P* < 0.0001). When all clones detected on days 1-3 were considered as true complexity of infection, sampling on day 1 detected only 69.4% (109/157) or 68.3% (228/334) of all clones in incident and chronic infections, respectively. Our findings demonstrate that a large proportion of clones are missed by single time-point sampling. In addition, because of the high complexity of infection early in incident infections, our data suggest many infections may be caused by genetically complex inocula.

The transmission of *Plasmodium falciparum* remains intense in many areas of sub-Saharan Africa. As a consequence, a considerable fraction of infected individuals in many settings may have multiclonal infections. Multiclonal infections may arise either from superinfection, whereby a single individual is infected with different parasites from multiple mosquito bites, or from cotransmission where a single mosquito transmits multiple parasite clones at one time. Repeated sampling to determine the complexity of infection (number of clones present during infection) indicates that a substantial proportion of clones may remain undetected when relying on single time point samples.^1^^–^^3^ The ability to detect all clones present during an infection is affected by fluctuating parasite densities, parasite sequestration patterns, and technical challenges such as competition in the polymerase chain reaction (PCR) reaction mix. Understanding the detectability of clones is relevant to appreciate to what extent single time point observations may be flawed. This is particularly important for drug efficacy studies when using genotypes on the day of treatment to determine whether an infection post treatment is recrudescence (treatment failure) or a new infection.^4^ Few studies have sampled naturally infected individuals for clonal complexity repeatedly over a short time window. In this study, we determined *P. falciparum* clones on 3 consecutive days in incident infections and chronic infections in Burkina Faso. Our hypothesis was that repeated sampling would increase the number of clones detected and that the detectability of clones would be lowest in chronic infections where parasite densities may be generally lower.

We addressed these hypotheses in a cohort of children aged 5–10 years, from Balonghin, Burkina Faso, an area exposed to intense malaria transmission. Informed consent was provided by the parent or guardian of each child. Ethical approval for the study was granted by the Ethical Review Committee of the Ministry of Health of Burkina Faso (Deliberation number 2015-3-033) and the Ethics Committee of the London School of Hygiene and Tropical Medicine (#9008). Full details of study cohorts are reported elsewhere.^5^ Briefly, the incident infection cohort included children (*N* = 42) who were parasite-free at the start of the transmission season by molecular methods and were monitored weekly by 18S nested PCR (nPCR)^6^ for incident infections. Once detected, infections were monitored daily. The chronic infection cohort included children (*N* = 54) who were parasite positive on at least two consecutive visits by nPCR that were spaced 4 weeks apart. Once this condition was met, daily sampling was performed as for incident infections. All individuals received treatment with the first-line antimalarial drug artemether-lumefantrine as soon as symptoms developed (measured axillary temperature ≥ 37.5°C or reported fever in last 24 hours). For the current study, only the first 3 days of intensive follow-up were considered. On those days, *P. falciparum* densities were measured by 18S quantitative PCR^7^ and clones were detected by PCR targeting the polymorphic marker gene merozoite surface protein 2 (MSP2) and capillary electrophoresis. Following automated sizing by PeakScanner^®^ software (version 2) using three basepair bins (Applied Biosystems, Thermos Fisher Scientific, Inc., UK), we applied a cutoff of 1,000 relative fluorescent units (RFUs) to conservatively distinguish true peaks from background signal and stutter peaks. All major peaks and any additional alleles with a minimum height of 10% of the major peak were scored. Complexity of infection (COI) was defined as the total number of clones detected on days 1–3 combined. The proportion of these clones that were detected either on day 1 of sampling, on combined days 1 and 2, or on combined days 1 and 3 was calculated for incident and chronic infections separately. The MSP2 allelic diversity was estimated by GeneAlEx version 6.5^8^ as equivalent to heterozygosity estimate. The association between parasite density and proportion of detected clones was assessed by a generalized linear model (GLM) with a logit link and the binomial family; parasite density was added to these models after log_10_ transformation. Analyses were performed in Stata 16.0 (Statacorp, College Station, TX).

When combining the 3 days of sampling, the total number of distinct parasite clones detected was 158 for the incident infection cohort and 335 for the chronic infection cohort. Among these clones, there were 105 (66.5%) distinct 3D7-specific alleles ranging in size from 207–460 bp and 53 (33.5%) FC27-specific alleles ranging in size from 209–424 bp for the incident cohort. For the chronic cohort, there were 217 (64.8%) 3D7-specific alleles ranging in size from 212–427 bp and 118 (35.2%) FC27-specific alleles ranging in size from 220–425 bp. The estimated haploid diversity for MSP2 was 0.981 in incident infections (with 88 detected alleles) and 0.976 in chronic infections (with 120 detected alleles). The median number of clones per infected individual was 3.0 (interquartile range [IQR] 2.0–5.0) for incident infections compared with 7.0 (IQR 4.0–8.0) in chronic infections (Mann–Whitney test, *P* < 0.0001), and this was underestimated with a single day of sampling (Table 1). All infections in the chronic infection cohort and 76.2% (32/42) of the incident infections were multiclonal (Figure 1A). Although this high COI may reflect the accumulation of clones in chronic infections over time, the data indicate that incident infections are also commonly multiclonal. Our intensive monitoring, with confirmed parasite negativity before incident infections, therefore, suggests that children experiencing incident infections were either bitten by multiple infected mosquitoes over a very short time period, which is plausible given the high mosquito exposure in some households in the study area,^9^ or they experienced genetically complex inocula from a single mosquito.^10^ Sampling on individual days considerably underestimated COI: approximately two-thirds of all present clones (day 1–3 combined) were detected on day 1 of sampling in both incident (69.0% 109/158) and chronic (68.1% 228/335) infections (Figure 1B). Interestingly, this level of detectability with 1 day of sampling was also observed in a similar study using the same genotyping method in a lower endemicity setting, Papua New Guinea.^2^ When samples were included from days 1 and 2, 82.3% (130/158) and 90.7% (304/335) of all clones were detected in incident and chronic infections, respectively; when including samples from days 1 and 3, these proportions were 86.7% (137/158) and 77.3% (259/335). Sampling on days 1 and 2 detected a similar proportion of all clones compared with sampling on days 1 and 3, with additional unique clones detected on day 3 that were not detected on day 2. Parasite density was a median of 676 parasites/µL (IQR 84–3,248) in incident and 545 parasites/µL (IQR 156–1,316) in chronic infections, and was strongly associated with the fraction of clones detected on the first day of sampling. With increasing total parasite density, the proportion of clones detected on day 1 increased (GLM model coefficient 0.70, 95% CI 0.41–0.98, *P* < 0.001). After adjusting for parasite density, the detectability of clones was not different between incident and chronic infections in our cohort (GLM model coefficient −0.41, 95% CI −1.03 to 0.22, *P* = 0.20).

**Table 1:** Clonal complexity in incident and chronic infections

	Incident infections (*N* = 42)	Chronic infections (*N* = 54)
Number of clones detected (*n*/*N* [%])		
Day 1	109/158 (69.0%)	228/335 (68.1%)
Day 2	21/158 (13.3%)	76/335 (22.7%)
Day 3	28/158 (17.7%)	31335 (9.3%)
Day 1 + Day 2	130/158 (82.3%)	304/335 (90.7%)
Day 1 + Day 3	137/158 (86.7%)	259/335 (77.3%)
Average complexity of infection (median clones per person [IQR])		
Day 1	2.0 (1–3)	5.0 (2–6)
Day 1 + Day 2	2.0 (1–4)	6.0 (4–7)
Days 1–3 combined	3.0 (2–5)	7.0 (4–8)

IQR = interquartile range.

**Figure 1.**
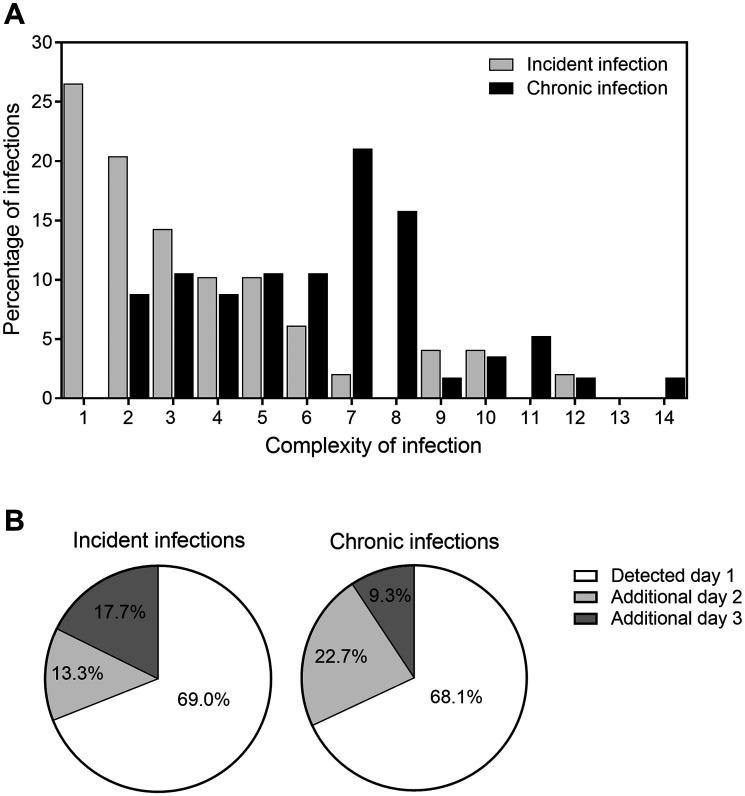
Detection of *Plasmodium falciparum* msp2 clones with repeated sampling on days 1, 2, and 3 in incident and chronic infections in Burkina Faso. (**A**) The distribution of complexity of infection (total number of clones detected on day 1, 2, and 3 for each individual) in incident and chronic infections. (**B**) The pie charts represent all clones detected in all participants per cohort on days 1, 2, and 3 combined. The sections of the pie chart indicate the proportion of all clones in all subjects per cohort that were detected on day 1 (white), the proportion of additional clones detected on day 2 but not day 1 (light gray), and the proportion of additional clones detected on day 3 but not on days 1 and 2 (dark gray).

In conclusion, our findings demonstrate that a large proportion of clones are missed by sampling a single time point. An additional sampling time point 24 hours later (i.e., days 1 and 2) or 48 hours later (i.e., days 1 and 3) greatly improved the detectability of clones, but either combination of 2 day sampling still missed between 9.3% and 22.7% of clones detected with 3 days of sampling. Since additional unique clones were detected on the third day of sampling, we conclude that parasite sequestration is unlikely to be the only explanation for missing parasite clones. Minority clones are difficult to detect^11^ and this complicates, for instance, reliably identifying antimalarial treatment failures if the parasite clone that survives treatment is present at low abundance at the initiation of the study. Application of novel, highly sensitive genotyping protocols, for example, amplicon deep sequencing, could help detect minority clones^12^ especially when repeated sampling is not possible.^13^ Our study was conducted in children residing in an area of high transmission intensity; this resulted in high COI estimates among study participants. Frequent sampling allowed us to detect infections early and, uniquely, investigate clonal complexity early-on in incident infections. The downside of this approach is that parasite densities were relatively low (resulting from early detection of infection by PCR) and this will have influenced detectability of clones. We also consider it plausible that sampling beyond day 3 would have increased the number of detected clones beyond our current findings. This would not affect any of the study conclusions but further highlight the complexity of accurately assessing infection clonal composition with sparse sampling approaches.
